# Supplementary material for: Are carbon emissions trading and green financial instruments synergistic? -Comprehensive quantitative research based on content analysis
Source: PLoS One. 2024 Mar 7;19(3):e0298601. doi: 10.1371/journal.pone.0298601 (PMC10919601; doi:10.1371/journal.pone.0298601)
Supplement: S1 Data — (ZIP) [file pone.0298601.s001.zip › Data/地级市Z_F工作报告.zip!/╣π╬≈/─╧─■╩╨/└┤╫╘╣1⁄2╓┌║┼í╛┬φ┐╦╩2╛▌═°í┐_2017.docx]

各位代表：

　　现在，我代表市人民政府向大会作政府工作报告，请予审议，并请各位政协委员和列席会议的同志提出意见。

　　一、2016年工作回顾

　　过去一年，面对错综复杂的发展环境，在自治区党委、政府和市委的正确领导下，我们紧紧围绕市委十一届十四次全会、市第十二次党代会的决策部署以及市十三届人大七次会议、市十四届人大一次会议确定的目标任务，认真践行五大发展理念，坚持稳中求进工作总基调，坚定信心、克难攻坚、精准施策、奋力拼搏，经济发展缓中有稳、稳中有进，社会和谐稳定，实现了“十三五”良好开局。

　　——经济发展稳中向好。地区生产总值完成 3703.39亿元、增长7%，规模以上工业总产值完成3537.05亿元、增长8.83%，固定资产投资完成3824.73亿元、增长13.6%，社会消费品零售总额完成1980.36亿元、增长10.84%，农业总产值完成689.03亿元、增长3.98%，外贸进出口总额完成416.23亿元、增长14.2%，财政收入完成613.83亿元、增长7.22%，实现了工业稳、投资稳、消费稳、农业稳、进出口稳、财政稳“六稳”目标。

　　——南宁进入地铁时代。全长32.1公里的地铁1号线全线通车，日均客运量超22万人次，不仅成为广西开通的第一条地铁，也是全国5个少数民族自治区开通的第一条地铁。2、3、4号线建设稳步推进。全年地铁完成投资123.45亿元。

　　——城市面貌日新月异。五象新区开发建设加速推进，年度完成投资301.8亿元，总部基地金融街高层建筑主体结构封顶达51.28%。民族大道改造提升全面完成。良庆大桥、沙井—南站立交等一批跨江桥梁和立交桥建成通车。那考河流域、南湖公园环湖路等一批海绵化改造项目顺利完成，海绵城市连片效应、生态治水成效初显，我市国家海绵城市试点做法获中央电视台《新闻联播》聚焦报道。

　　——“南宁蓝”成为常态。持续加大节能减排工作力度，深入开展大气污染防控特别是扬尘污染专项治理，空气质量优良率逐年提高，2016年达95.1%，同比上升6.3个百分点，4次入围全国重点城市月度评比十佳，南宁蓝天常驻。

　　——“十三五”脱贫攻坚首战告捷。邕宁区和120个贫困村顺利摘帽，12.8万贫困人口实现脱贫。

　　——利民惠民取得新成效。全年民生支出433.95亿元、增长13.02%，占一般公共预算支出73.92%。全市居民人均可支配收入22862元、实际增长7.4%。居民消费价格上涨1.4%。城镇新增就业7.82万人，城镇登记失业率控制在2.62%。全面完成区、市共76项为民办实事工程项目。

　　一年来，我们突出做好以下六项重点工作：

　　（一）着力推动产业转型升级，重质量、增效益

　　以创新驱动为引领，发展新经济培育新动能，推动了发展方式加速转变、中高端产业加速形成、产业结构加速优化。

　　加快发展新模式。推进大众创业万众创新，南宁·中关村双创示范基地正式运营，哈工大机器人、上海明匠等18家行业领军企业入驻，国际孵化器雨林空间成功引进19个初创团队，基地辐射带动效应初步显现。68个创客团队和8家服务机构入驻南宁创客城，形成以南宁软件园、中盟科技园为核心的现代科技企业孵化产业集群。“四众”新模式加快发展，国家和自治区级备案创客空间5个，新增入孵企业61家。

　　加快形成新动能。全力推进150项重点工业项目建设，柳药中药饮片生产基地（一期）、华润怡宝健康饮品广西生产基地等项目竣工投产，科天水性科技产业园、南南电子汽车新材料精深加工等项目开工。电子信息、先进装备制造、生物医药三大重点工业产业完成产值1329.51亿元、增长16.3%，占全市工业比重达37.59%。重点打造富士康千亿元产业园，富士康南宁公司年产值超320亿元；南南铝加工公司自主研发的航空航天、轨道交通高端铝材产品达到国际先进水平。加快培育石墨烯、新能源汽车等新兴产业，广西首家石墨烯研发企业落户高新区；源正新能源汽车客车整车下线，中车轨道装备基地实现地铁车辆制造，南宁公交南宁造、南宁地铁南宁造实现历史性突破。产值超亿元工业企业633家，拉动全市规模以上工业总产值增长11个百分点。实施金融业、现代物流业、电子商务和信息服务业“三年行动计划”，重点扶持12个现代服务业集聚区建设，第三产业对经济增长贡献率达60.9%。广发银行南宁分行开业运营，新增上市和新三板挂牌企业11家，金融业增加值完成405.8亿元，对经济增长贡献率达14.5%。A级物流企业达15家，太华现代医药物流等项目建成使用，开通以南宁为货运物流中枢的东盟—中欧国际列车公铁联运新通道。软件和信息技术服务业实现主营收入120.24亿元、增长17.8%，总量占全区80%以上。

　　加快培育新业态。自媒体运营、移动支付、网上商城、体验式消费传播等新业态快速成长。大力扶持300家企业在第三方电商平台开设网店，重点企业电子商务交易额达2201亿元、增长22%。南宁跨境贸易电子商务综合服务平台不断完善。南宁跨境商品直购体验中心顺利开业。亿元税收经济楼宇达10栋。全市新增限额以上商贸企业214家，限额以上消费品零售总额增长9.96%。完善县乡村三级农村电商服务体系，县级电商服务中心达6个、农村电商产业园达7个、村级服务点（体验店）超1100个，横县、宾阳获批全国电子商务进农村综合示范县。“三品一标”认证农产品新增14个、总数达137个，“刘圩香芋”“那楼淮山”获国家农产品地理标志登记保护。坚持产村互动、农旅融合，推进“示范区+基地+农户”等经营模式，新建的12个市级现代特色农业示范区引进企业34家，5个示范区被认定为第三批广西现代特色农业示范区。推进智慧旅游，全市接待旅游总人数、旅游总消费分别突破9000万人次、900亿元，分别增长16.38%、23.72%，入围国家全域旅游示范区创建市，上林成功创建广西特色旅游名县。

　　（二）着力服务项目和企业，促投资、稳增长

　　精心服务项目和企业，扩大投资有效性，提升企业竞争力，发挥投资对稳增长的关键作用。

　　“简”审批环节。扎实做好行政审批“放管服”各项工作，年度共取消和调整行政审批事项96个。加快市行政审批局实质性运转。三大国家级开发区行政审批再提速。清理规范行政审批中介服务事项99项。建立完善全市统一的网上审批大厅，38个部门412项政务服务事项实现网上申办预审服务，项目审批提速增效。创新建立政府部门权责清单“三个一”模式，乡镇政府（街道办）权责清单已向社会公布，解决服务基层群众“最后一公里”问题。我市行政审批制度改革经验获中央电视台《新闻联播》聚焦报道。

　　“真”服务企业。坚持市领导联系重大项目服务重点企业制度，开展“千名干部入千企”活动，打好服务企业 “组合拳”。强化融资服务，人民币新增贷款1195.13亿元，1305家企业通过南宁股权交易中心实现挂牌、融资4.35亿元，15家上市企业通过资本市场累计融资360.26亿元，296家中小微企业加入“市助保贷重点中小企业池”，“两台一会”为316家企业投放贷款32.82亿元。强化人才服务，资助高层次创业创新人才团队3250万元，实施“百名工科博士硕士入邕企”等项目、引进高层次人才45名。主动帮助劳动密集型企业解决用工荒问题。成功争取自治区将直冠“广西”企业名称核准权下放我市各县区。新增广西名牌产品32个，15家企业荣获广西服务业品牌称号，获批创建全国质量强市示范市。

　　“优”投资结构。全年实施项目10400个、竣工项目7582个。501个区市层面统筹推进重大项目完成投资813.16亿元。以电子信息、医药制造为代表的先进制造业完成投资301.93亿元、增长8.12%，占二产投资29.29%。以信息服务、科技服务为代表的现代服务业完成投资818.9亿元、增长24.72%，占三产投资30.92%。房地产开发投资完成854亿元、增长29.95%，拉动全市投资增长5.85个百分点。民营经济加快发展，民间投资增长15.64%，占全市投资的64.34%；19个PPP项目加快推进，5个入选财政部示范项目；新增个体户6.38万户、私营企业4.48万户，其中第三产业新登记企业占总数的88.26%。形成以中央预算内资金、专项建设债、政府投资基金、企业债、PPP等融资方式的新局面，推动总规模为830亿元的南宁城市发展基金落地。

　　（三）着力提升生态宜居水平，强基础、优环境

　　坚持治水、建城、为民主线，推动“五个融合”，全力打造高品位的生态宜居城市。

　　推动水绿融合。实施国家海绵城市试点项目142个，完成投资50.3亿元，累计建成区域面积达25.2平方公里。邕江综合治理核心区域33公里两岸河段全线开工建设，邕江自治区党校至三岸大桥段完工，57.5公里河道船只完成专项清理，邕江水环境整体提升。全面完成297个生活污水直排口整治，从源头削减水体污染源。消除或基本消除朝阳溪等约43公里河段黑臭水体，完成了国家“水十条”对城市黑臭水体治理的年度考核任务。实施大王滩水库、龙潭水库环境综合整治，保护库区水生态环境。成功申办第十二届中国国际园林博览会，荣获首批“国家生态园林城市”。深入开展“美丽南宁·生态乡村”活动，完成自治区级绿化示范村屯447个、一般村屯5714个，马山成为国家第二批生态文明先行示范区。

　　推动产城融合。有序开展新一轮城市总规修编，《南宁空间发展战略规划》等22项重点规划和专项规划编制完成。统筹做好“多规合一”，初步形成GIS“一张图”。推进永久基本农田划定，调整完善土地利用总体规划。推动产业集聚园区化、园区建设城市化，三大国家级开发区完成规模以上工业总产值占全市比重达54.11%，拉动全市规模以上工业总产值增长8.66个百分点。全市建成区面积287.37平方公里，常住人口城镇化率达60.23%。

　　推动新旧融合。五象新区国家级新区申报工作加紧推进，新区在建项目471个、增长22.02%，在建项目面积2436万平方米、增长24.22%，邕宁新兴产业园等园区建设加速；机场第二高速、玉洞大道改扩建等主干道如期建成通车，片区路网建成通车道路达53条，三中五象校区、五象新区第一实验小学等4所优质学校如期建成招生，医院、邻里中心、供水加压站等一批设施项目加快建设或建成使用；创新实施“房票”安置新模式，安置工作由“建房”转向“上房”阶段。全市 “三旧”改造步伐加快，完成11个项目的土地熟化人征集工作，投资100亿元；棚户区改造开工1.98万套，货币化安置5057户；完成国有土地房屋征收面积57.31万平方米；“老南宁·三街两巷”改造有序推进；西乡塘片区基础设施建设加快，逐步形成新老城区协调发展格局。

　　推动建管融合。安排城建计划项目989项，完成投资402.7亿元。完成市政道路“白改黑”工程36条。轨道交通线网规划修编及第三轮建设规划启动。首条快速公交线路开通运营，国际会展中心改扩建、凤凰岭路改扩建等重大项目顺利完成。南宁东等5个高速公路收费站外迁，城市发展空间进一步拓展。完成449个千人以上农村集中供水工程饮用水水源地保护区划定工作，邕江5个集中式饮用水源地水质、主要流域水环境功能区达标率均为100%。实施农田水利工程624项，新增、恢复改造灌溉面积26.34万亩。加强环境联防联控，推进水、大气、土壤治理。城南垃圾填埋场封场，平里静脉产业园启用，餐厨废弃物资源化利用和无害化处理“南宁模式”荣获第三届广州国际城市创新奖，南宁成为国内两个获奖城市之一。城市生活垃圾无害化处理率保持100%。启动农村垃圾专项治理两年攻坚战，农村人居环境明显改善。推进全国综合行政执法体制改革试点，推广“智慧青秀”APP经验，打造“互联网+城管”，推进城市管理精细化。

　　推动“里”“面”融合。入选全国地下综合管廊试点城市，开工建设项目12个、投资5.48亿元，广西首条综合管廊佛子岭路综合管廊投入使用，逐步解决“马路拉链”“空中蛛网”问题。市本级累计建成污水管网112公里，埌东污水处理厂水质提标改造工程完工，出水水质达一级A标。加强城市设计，注重城市建筑及景观风貌的美观适用，推进城市修补和生态修复。深入开展“美丽南宁·整洁畅通有序大行动”，查处各类“五乱”案件105.9万起，清理违法占地面积256.73万平方米，拆除违法建筑面积186.44万平方米。深化拓展 “礼让斑马线”主题活动，打造首府文明新品牌。

　　（四）着力推进改革创新，添动力、激活力

　　坚持问题导向，不折不扣落实各项改革措施，为经济社会发展添动力激活力。

　　扎实推进供给侧结构性改革。千方百计降低实体经济成本。出台30条措施，从融资、税费、物流以及制度性交易成本等方面为各类企业降本减负39亿元。阶段性降低社保费率，为企业减负4.93亿元。升级优化传统产业。启动92家国有“僵尸企业”清理工作，完成淘汰钒冶炼、平板玻璃等去产能任务，改造提升低效电机4.23万千瓦。打通供需通道去库存。财政补贴1.76亿元，鼓励非南宁市户籍居民家庭购买首套新建普通商品住房2.93万套，落实调整房地产交易环节契税和营业税（增值税）政策，新建商品房去化周期为11个月。积极稳妥防风险。实施政府债务限额管理，推进政府存量债务转换为债券，获自治区发行转贷政府债券资金240.64亿元。

　　统筹推进重点领域改革。承担的自治区20项重大改革任务和41项国家、自治区级试点建设扎实推进，市本级159项改革任务基本完成。深化商事制度改革，实施企业“六证合一”，在全区率先启动“双随机、一公开”联合检查和企业简易注销改革。全面完成不动产登记“发新停旧”工作，实现“房地合一”。建成网上行政执法暨电子监察系统，完成城市管理、农业、交通运输领域综合执法试点。加快推进市县两级公共资源交易平台整合。成立市财政预算绩效管理局，将绩效目标管理贯穿于财政预算全过程。顺利实现全面营改增税制转换。建立完善现代企业制度，完成一批企事业单位公司化、股份制改制，推进混合所有制、职业经理人等多项国企改革试点。深入推进沿边金融综合改革试验区建设，跨境人民币结算量202.54亿元。行政村建立“三农”金融服务室覆盖面达45%。率先在全区探索建设统一土地承包经营权确权登记信息管理系统。深化供销社综合改革。乡镇“四所合一”改革实现全覆盖。推进统一社会信用代码制度，完善失信惩戒制度。

　　不断完善创新体系。开展科学研究与技术开发计划项目413项、总投资12.04亿元。新增广西创新型企业7家、自治区级重点实验室和工程技术研究中心4家。实施发明专利双倍增计划，全市发明专利受理量1.32万件，每万人口发明专利拥有量6.72件，比上年增2.01件。高新技术企业达304家，高技术产值增长14.48%；博世科、田园生化被认定为全国技术创新示范企业。博世科参与研发的“造纸与发酵典型废水资源化和超低排放关键技术与应用”项目获国家科技进步二等奖。市“两化”融合公共服务平台及体验中心一期挂牌运营，广西中烟、中建泓泰入选国家工信部第三批“两化”融合管理体系贯标试点。

　　（五）着力扩大开放合作，建平台、提水平

　　积极融入“一带一路”、中国—东盟自贸区升级版等建设，提升“南宁渠道”影响力。

　　突出平台载体建设。圆满服务第十三届中国—东盟博览会和商务与投资峰会，成功举办或承办2016亚洲国际集邮展、第九届泛北部湾经济合作论坛暨中国—中南半岛经济走廊论坛等重大活动。国际友城达21个，荣获全国国际友好城市交流合作奖。中国—东盟信息港南宁核心基地建设全面启动，并获批为自治区重点产业园区，中国移动五象信息交流中心等项目加快推进，广西东盟信息交流中心一期工程等竣工。南宁综合保税区一期顺利通过验收，具备封关运营条件。空港经济区成功引进邮政陆运及跨境电商中心等6个产业项目，空港保税物流园启动建设。

　　提升互联互通水平。区域性国际综合交通枢纽建设有新突破，吴圩机场第二跑道和综合交通换乘中心前期工作加快推进，伶俐通用机场开工建设；南昆客专开通运营，火车西站建成使用，火车东站成为我国南方最大的综合性交通枢纽之一，南昆铁路南宁至百色段增建二线、黎湛铁路电气化改造等建设加快，贵南客专开工建设；吴圩机场至大塘高速、贵隆高速、柳南高速改扩建工程建设加快；老口航运枢纽顺利竣工，邕宁水利枢纽加快推进，西津二线船闸开工。面向东盟跨境物流甩挂运输试点项目有序推进。广西—东盟区域（南宁）外贸一体化综合体通关服务提速工程实现试运营，阿里巴巴“一达通”等外贸综合服务企业落户南宁，贸易便利化水平不断提升。

　　加快发展外向型经济。深入实施招商引资三年行动计划和外经贸发展三年行动计划，深化与粤港澳台合作，加快加工贸易梯度转移重点承接地、中国服务外包示范城市、CEPA示范城市建设。全市加工贸易进出口额272亿元，增长11.8%。服务外包合同执行额2.59亿美元，增长96%。实现对外投资额8.2亿美元，增长10.8%。围绕重点领域重点产业精准招商，全年区外境内实际到位内资682.9亿元，全口径实际利用外资7.7亿美元，分别增长11.7%、9.8%。

　　（六）着力保障改善民生，保基本、促和谐

　　牢固树立以人民为中心的发展思想，坚持普惠性、保基本、均等化、可持续方向，利民惠民，增进人民福祉。

　　全面完成年度脱贫攻坚任务。大力推进“七个一批”“七大工程”。全市筹措整合各类涉农资金80.76亿元投入脱贫攻坚。强化驻村帮扶和定点扶贫，实现贫困村党组织第一书记、扶贫工作队员全覆盖。扶持优势特色产业项目1429个，引进、培育新型经营主体941个。帮助贫困劳动力转移就业1.19万人次、扶持创业1066人。开工建设移民安置点21个。生态扶贫1629人。资助建档立卡贫困户学生16.2万人次、资金1.48亿元。推进农村低保制度与扶贫开发政策有效衔接，医疗和临时救助贫困人口7223人次、资金1281.8万元，贫困人口新农合参合率98.8%。4929名贫困残疾人享受残疾人托养服务和补助资金739.35万元。修建贫困村屯级道路998.91公里。实施贫困村饮水安全项目225处，11.15万人受益。竣工农村电网改造升级项目1984个。完成建档立卡贫困户农村危房改造7201户。

　　各项社会事业协调发展。推动社会主义核心价值观深入人心，“南宁好人馆”建设启动。“全面改薄”“双千计划”扎实推进，新建成并投入使用中小学校13所，改扩建学校1030所，学前三年毛入园率95.3%，九年义务教育巩固率96.02%，高中阶段毛入学率95.13%；西乡塘、武鸣、马山通过全国义务教育发展基本均衡县区国家督导评估认定；突出抓好特殊教育、农村留守儿童教育；全年共接收进城务工人员随迁子女入学约13万人；职业教育服务地方经济发展能力进一步增强；南宁学院通过自治区学位委员会学士学位授权评估；南宁教育园区基础设施完成投资9.9亿元，新签约入驻院校13所。深入实施“全民参保登记计划”，城乡居民基本养老保险参保率93.29%，全市社会保险参保达676.67万人次（不含新农合）。农村低保保障标准增幅达12.9%，城市低保平均保障标准达467元/人·月。基本建成各类保障性住房2.36万套，分配入住1.79万套，新增发放租赁补贴2767户。入选全国健康城市试点，全国养老服务业综合改革试点扎实推进，列入国家医养结合试点城市，建成一批城乡养老服务机构。启动城市公立医院综合改革，新增医疗服务机构209家、床位1346张。加强基层医疗卫生机构能力建设，基层医疗服务和保障水平不断提升。稳妥推进全面两孩政策实施，人口计生各项约束性指标控制在指标范围内。实施体育场地建设、改造项目335个，经常参加体育锻炼人数比例达46%，成功承办首届“中国杯”国际足球锦标赛等重大体育赛事12项。新建村级公共服务中心125个；打造民歌湖“百姓大舞台”周周演群众文化活动品牌，各类惠民演出超4000场；加强顶蛳山遗址等文化遗产保护传承；“春天的旋律”跨国春晚入选“2016国家丝绸之路影视桥项目”25强，成功举办中国—东盟（南宁）孔子文化周、中国—东盟（南宁）戏剧周等，对外文化交流影响力扩大。文化产业不断壮大，一批文化骨干企业成功登陆新三板，《漂移岛之天空历险记》成为首部登陆央视的桂产动画片。

　　加强和创新社会治理。深入推进法治南宁、平安南宁建设，全面推进社区网格化管理，加快社会治安防控体系建设，抓好反恐防暴和维护政治安全、公共安全、网络安全等重点领域风险防控工作，严厉打击电信诈骗等各类违法犯罪活动，社会治安呈现“九降九升”的良好态势。初步建成市县（区）乡（镇、街道）三级公共法律服务体系。实行诉访分离，畅通信访渠道，加大社会矛盾纠纷调处化解力度，公众安全感和满意度稳步提升。国家食品安全城市创建试点工作稳步推进，食品药品安全监管能力有效提升，武鸣荣获第一批“国家农产品质量安全县”。严格落实安全生产“党政同责、一岗双责、齐抓共管、失职追责”，无重特大生产安全事故发生。

　　此外，国防教育和后备力量建设深入开展，荣获“全国双拥模范城”六连冠、“自治区双拥模范城”八连冠。民族宗教、人防等工作取得新成效，荣获“全国民族团结进步创建活动示范市”“全国人民防空先进城市”。统计、侨务、水库移民、地震、机关事务管理、地方志、保密、档案、出版、消防、海关、海事、检验检疫、税务、调查、测绘、气象、水文、参事、文史、哲学和社会科学等工作不断加强，对台工作取得新成绩，妇女、儿童、老年人、残疾人等事业健康发展。

　　2016年，我们深入开展“两学一做”学习教育，加强党风廉政建设，严查发生在群众身边的“四风”和腐败问题，强化扶贫领域监督执纪问责，促进政府系统廉政勤政。全面履行政府职能，强化依法行政，推进行政权力在阳光下运行，提请市人大常委会审议地方性法规草案2件，出台政府规章3件、规范性文件46件。建立市政府例行新闻发布制度。全年公开政务信息14.1万条。启动“七五”普法工作。坚决落实中央八项规定精神，严守国务院“约法三章”，严控行政经费增长，“三公”经费压缩32.58%，加强对重点领域、重点资金、重点部门的审计，举办电视问政直播节目10期、131个问题全部整改。认真执行市人大及其常委会的决议、决定，自觉接受市人大及其常委会法律监督和工作监督，主动接受人民政协民主监督和社会舆论监督，办理国家、区、市三级人大代表议案和建议234件、政协提案316件，办结率100%，并荣获自治区政协2016年度提案先进承办单位。同时，连续6年被评为人民网网友留言办理工作先进单位，市长留言回复率在全国地市级层面位列第一。

　　各位代表，一年成绩来之不易。这是自治区党委、政府和市委正确领导的结果，也是全市各族人民同心同德、顽强拼搏的结果。在此，我代表市人民政府，向全市各族人民，向人大代表、政协委员，向各民主党派、工商联、无党派人士、各人民团体和各界人士，向驻邕人民解放军、武警部队官兵，向所有关心支持南宁建设发展的港澳台同胞、海外侨胞及国际友人，表示衷心的感谢并致以崇高的敬意！

　　各位代表，在肯定成绩的同时，我们也清醒地看到发展中的困难和问题：多项经济指标增速回落；民间投资增长乏力，新旧动能转换步伐不快，工业增长缓慢，金融业增加值增速降幅较大；城乡发展不够协调，县区经济增长乏力；脱贫攻坚任务繁重，不少民生诉求还未得到有效解决；政府系统少数干部不敢担当、不愿作为，等等。这些问题，我们要勇于正视，直面困难，切实研究解决。

　　二、2017年工作安排

　　2017年是党的十九大召开之年，是全面贯彻落实自治区第十一次党代会、市第十二次党代会精神的开局之年，也是迎接自治区60周年大庆的关键之年。我们将全面贯彻落实党的十八大和十八届三中、四中、五中、六中全会以及中央经济工作会议精神，深入贯彻习近平总书记系列重要讲话精神和治国理政新理念新思想新战略，紧紧围绕自治区第十一次党代会、市第十二次党代会和市委十二届三次全会决策部署，统筹推进“五位一体”总体布局和协调推进“四个全面”战略布局，坚持稳中求进工作总基调，牢固树立和贯彻落实新发展理念，适应把握引领经济发展新常态，以提高发展质量和效益为中心，以推进供给侧结构性改革为主线，全力稳增长、促改革、调结构、惠民生、防风险，加快推进“六大升级”工程，为加快建设“四个城市”，勇当广西营造“三大生态”、实现“两个建成”的排头兵打下坚实基础，以优异成绩迎接党的十九大胜利召开。

　　经济社会发展主要预期目标是：地区生产总值增长7.5%左右，财政收入增长6%，固定资产投资增长11.5%，社会消费品零售总额增长10%，规模以上工业增加值增长7.5%，进出口总额增长8%，节能减排降碳控制在自治区下达目标内，全市居民人均可支配收入实际增长7.5%，居民消费价格涨幅控制在3%以内，城镇登记失业率控制在4%以内，城镇新增就业6.5万人。

　　为实现上述目标，2017年，我们重点抓好以下工作：

　　（一）大力推动实体经济发展和产业转型升级

　　坚持创新驱动发展，大力实施质量品牌战略，加快发展新技术、新模式、新业态，推动产业发展迈向中高端。

　　深入实施创新驱动发展战略。全面深化科技体制改革，改进财政科技经费投入管理。加强知识产权保护力度，推进发明专利双倍增计划。实施科技成果转化行动，完成科技成果转化56项。鼓励企业加大创新投入，新增市级以上企业技术中心6家。加强品牌培育，培育2家自治区级以上质量管理标杆企业、25个广西名牌产品，培育和申报1个以上国家地理标志产品。积极创建国家自主创新示范区，实施高新技术企业倍增计划和“瞪羚企业”培育计划，新增高新技术企业36家，入库培育企业40家。年内完成200家企业的“两化”融合评估诊断和对标引导，2家企业通过国家“两化”融合管理体系评定。发挥南宁·中关村双创示范基地的辐射作用和溢出效应，加快入驻企业的创新成果转化，全力申报国家级双创示范基地，努力打造一个领军人才、核心技术、高端产业集聚的平台；加快提升南宁创客城、创客空间等平台的服务功能，孵化出一批新点子、新企业、新产品，努力建设一个“草根”创业创新的载体；培育南南铝加工研究院、富士康东盟硅谷科技园等创新平台升级为国家级研发机构。紧扣产业链和创新链，加强创新型人才队伍建设。

　　推进工业转型发展。做大做强三大重点产业。以重点企业为龙头，着力延长产业链，加快打造千亿元产业集群，力争三大重点产业产值占全市工业比重达38%以上。电子信息产业方面，以高新区、江南工业园等为平台，依托中国—东盟信息港南宁核心基地、富士康千亿元产业园等载体建设，大力发展中高端电子信息产品制造，加快十一科技电子信息产业园等项目建设，力争禾田信息港等项目投入运营；大力引进上下游产业和相关配套产业，加快形成以富士康等为龙头的电子信息产业集群；促进大数据、云计算、物联网等智慧产业集聚发展，力争电子信息产业完成产值530亿元。先进装备制造产业方面，以邕宁新兴产业园、江南工业园、高新区、经开区等为平台，加快南南电子汽车新材料精深加工、源正新能源汽车项目建设，大力引进上下游配套企业，加快打造面向西南和东盟市场的南宁新能源汽车生产基地；以研祥智谷、中车轨道城轨车辆、中车精密列车大部件、中铁广发盾构机项目为龙头，打造西南最大的轨道交通装备产业集群；以南南铝加工为依托，重点引进铝制汽车、城轨机车、城市铝制设施、电子铝材等铝深加工及下游产业项目，打造特色高端铝精深加工产业园；以哈工大机器人、上海明匠等智能制造领军企业为支撑，积极发展机器人、无人机等高端制造业，推动我市制造业向智能化制造转型升级，力争先进装备制造产业完成产值780亿元。生物医药产业方面，抓好经开区生物医药产业园、南宁高新技术生物产业基地等平台建设，以现代中药、生物制药、高端医疗器械等为主攻方向，加快推进修正健康产业基地、诺博医疗移动终端设备生产、培力药业异地改造、百会药业等项目建设，推动海王保健品产业园等项目实现投产，促进柳药一期等项目达产；积极引进国内外医药大企业，加快完善医药物流配送网络，力争生物医药产业完成产值160亿元。培育发展新兴产业优化传统产业。重点发展新材料、节能环保、新能源等新兴产业，加快石墨烯研究成果产业化，推进科天水性科技产业园、博世科环保产业高安基地迁建等一批项目建设，提高新兴产业对工业的贡献率。加快改造提升食品加工、化工、建材等传统优势产业，全面推进BIM技术应用和装配式建筑发展，促进建筑业稳定健康发展；推进传统产业研发、生产、管理等模式变革，焕发传统产业新生机。深入实施园区经济倍增跨越计划。加快园区基础设施建设。深化开发区管理体制改革，积极发展“一区多园”，拓展发展空间，有序推动国家级开发区统筹城区工业发展。全市工业园区规模以上工业总产值占全市比重达85%以上，三大国家级开发区规模以上工业总产值平均增长20%以上。

　　增强服务业优势。以服务业集聚区建设为抓手，深入实施金融业、现代物流业、电子商务和信息服务业 “三年行动计划”，加快发展旅游、文化、体育、健康、养老五大幸福产业，推动生产性服务业向专业化和价值链高端延伸、生活性服务业向精细化和高品质转变。把金融业摆在更加突出的位置。完善金融组织体系，加快五象新区总部基地金融街、青秀区金湖金融中心、东盟商务区互联网金融产业基地等集聚区建设；争取平安银行等在我市设立分支机构；培育发展地方法人金融机构，推进南宁农商行组建工作。吸引境内外资本设立股权投资基金，配合自治区在我市设立合资证券公司，加快推动华润基金等实质性落地，做大做强南宁金融投资集团。力争全市金融业增加值达440亿元。提升现代物流业发展水平。加快冷链物流建设，优化功能区布局，增强园区集聚功能，完善吴圩空港物流园、牛湾港区综合物流园等园区建设，加快推进南宁云鸥物流食糖仓储智能配送中心等项目建设，重点扶持3A级以上物流龙头企业发展。加快发展电子商务和信息服务业。实施“电商南宁”战略，推进国家电子商务示范城市建设，加快南宁市跨境贸易中心、中国—东盟（南宁）跨境电子商务产业园等集聚区建设，扶持电商企业发展，培育电商品牌，将电子商务产业培育成为经济增长新亮点。完善信息消费基础设施建设，大力发展服务外包、软件研发、公共和商业信息等信息服务业。大力发展旅游业。积极推动我市及上林争创国家全域旅游示范市（区），马山、邕宁创建广西特色旅游名县，推进旅游业与其他产业融合发展。抓好百里秀美邕江、南宁国际旅游中心等重大旅游项目建设。推进大明山等创建国家5A级旅游景区，打造青秀山—万达茂—东盟文化博览园等环青秀山都市休闲精品线路，打造国际养生休闲旅游目的地。加快发展大健康产业。全面放开养老服务市场，推动国家医养结合试点城市建设，发展“互联网+养老”。支持开发和推广大众运动养生等健康产业，推进全民健身和全民健康深度融合。保持房地产市场平稳健康发展。坚持“房子是用来住的，不是用来炒的”定位，加强分类调控，调整土地供应结构，推进城镇低效用地再开发，提高土地利用效率和综合承载能力，保持市场供需平衡。加强房地产市场监管，强化商品房预售管理和商品住宅价格监管，规范房地产开发、销售、中介等行为。大力培育和发展住房租赁市场，推进房产交易管理和不动产登记工作深度融合。着力推进系统互通、信息共享的“智慧房管”平台建设。加快建立住房保障和房产管理领域信用管理制度。推广小区智能化管理，全力抓好物业服务行业监管。

　　推进农业特色发展。深入推进农业供给侧结构性改革，把增加绿色优质农产品供给放在突出位置，狠抓农产品标准化生产、品牌创建、质量安全监管工作，促进农业增效、农民增收。发展高品质特色农产品。深入实施“10+3”特色农业产业提升行动，打好生态牌、绿色牌、富硒牌，新建蔬菜、畜禽、水果等高标准“菜篮子”基地60个以上，新建规模化蔬菜基地面积4000亩以上；新建富硒农产品基地5个、面积2.5万亩；引导糖、蔗一体化生产发展，新增“双高”糖料蔗基地16.5万亩；新增“三品一标”认证农产品10个；深入实施种子工程和畜禽良种工程，高标准建设良种繁育基地10个；推动现代特色农业示范区扩面提质，力争申报自治区级示范区6个。培育发展农业新业态。扎实推进“互联网+农业”，落实与阿里巴巴等企业签署的合作协议，抓好全国电子商务进农村示范县试点，力争电商覆盖率60%以上；加快邮政系统服务“三农”综合平台和农产品产销联盟建设；探索建立优势农产品竞价交易模式；加快建设一批特色农林产品生产加工示范基地，加大农产品冷链基础设施建设力度；启动休闲农业示范区建设，打造3条农业与乡村旅游精品线路，促进农村一二三产业融合发展。深化农村综合改革。推进农村土地“三权分置”，推进农村土地承包经营权确权登记管理信息系统建设和有效运转；基本完成全市农村土地承包经营权确权登记颁证工作，推进武鸣农村承包土地经营权抵押贷款试点；加快农业经营机制创新，新增农民专业合作社500家、家庭农场150家。夯实农业发展基础。严格落实耕地保护制度，强化耕地占补平衡责任，完成永久基本农田划定上图入库，推进耕地提质改造工程，粮食播种面积稳定在650万亩以上。推进农业绿色发展，大力发展生态循环农业，扩大稻田生态种养规模，全面推广以“微生物+”为核心的现代生态养殖模式，完成30%的畜禽规模养殖场生态化改造。完善农业保险制度，主要农产品政策性农业保险参保率达35%。加强农产品质量安全监管，推进农产品质量安全从田头到餐桌可追溯管理。

　　（二）全力扩大有效投资

　　全面落实国家系列促投资政策，强化用地、资金等要素保障，努力增加有效投资。

　　狠抓重大项目建设。力争年内区市层面统筹推进重大项目完成投资700亿元以上。广西国际壮医医院等迎接自治区成立60周年重点项目完成投资190亿元以上。第一批城建计划实施项目889个、投资518亿元。

　　促进工业投资加快回升。重点加快推进100项新开工项目和50项续建项目，推进南宁浮法玻璃公司、南南铝业公司等老企业整体搬迁。落实重点工业产业扶持政策，通过资金补助、奖励、产业基金投资等多种方式，吸引骨干龙头企业进驻我市。鼓励企业零土地技改，重点加快八菱科技乘用车中冷器生产线技术改造、燎旺车灯生产基地技改等重点工业企业的产品升级、智能化改造项目。

　　激发民间投资活力。降低准入门槛，优化服务，促进民间投资。全力推进PPP项目25个，力争落地7个。继续做好企业上市（挂牌）培育和协调服务，推动更多的企业实现IPO上市或新三板挂牌。鼓励上市企业再融资，支持上市企业开展并购重组和产业整合。积极推动南宁创业投资引导基金、城市发展基金、产业发展基金和北部湾经济区产业基础设施投资（南宁）引导基金的运作，拓宽融资渠道。开展民间融资登记服务机构试点。加大对南宁再担保公司、“两台一会”、南方担保公司等支持力度，努力解决中小微企业融资难问题。加大对民营经济发展的政策支持力度，鼓励民营经济参与国企改革，增强企业投资信心。

　　保持基础设施投资强度。民航方面。配合推动吴圩机场军民分离和基础设施建设，力争开工建设吴圩机场第二跑道和综合交通换乘中心，推动T3航站楼前期工作，加快建设伶俐通用机场。铁路方面。完成南昆铁路南宁至百色段增建二线、黎湛铁路电气化改造项目建设，积极配合推进贵南客专等铁路项目建设，推动五象火车站、湘桂铁路扩能南宁至崇左段项目前期工作。公路方面。加快推进柳南高速改扩建工程、吴圩机场至大塘高速、贵隆高速以及柳州经合山至南宁第二高速公路等项目建设。黄金水道方面。推进西津二线船闸、南宁港旅游码头等项目建设，开工建设南宁港区锚地、隆安港区浪湾作业区、六景港区鹤笋作业区等。客运枢纽方面。加快推进凤岭综合客运枢纽站等客运枢纽项目。路网建设方面。推进轨道交通1号线西段沿线道路整治提升工程、凤岭北路（快环—高坡岭）等182个项目建设。

　　全力服务企业发展。实施大型骨干企业培育工程，支持企业间战略合作和跨行业、跨区域兼并重组，培育壮大一批竞争力强的大企业集团。扶持本土企业发展。扶持处于种子期、起步期的企业以及中小微企业发展。引导企业树立质量第一的强烈意识，发扬“工匠精神”，增强产品竞争力。进一步完善领导服务企业工作机制，构建“亲”“清”新型政商关系。

　　（三）不断扩大“南宁渠道”影响力

　　按照自治区“四维支撑、四沿联动”的布局，着力建平台、强合作、扩贸易，加快推动“南宁渠道”升级。

　　突出“1+3”开放平台建设。积极融入“一带一路”，积极配合打造中国—东盟博览会升级版，推动开展中国—中南半岛经济走廊试点示范工作，全力服务好第十四届中国—东盟博览会和商务与投资峰会。争取五象新区获批建设国家级新区，加快构建“一核、两廊、五区”的总体布局，努力打造我市加快开放发展的核心平台。

　　加快中国—东盟信息港南宁核心基地建设，重点推进启迪东盟科技城、广西电子政务外网云计算中心等项目建设。推动南宁综合保税区高效运营，加强与区内各沿边、沿海口岸海关等部门的联动合作，一期开发建设达35%，同步开展二期和配套区规划建设。加快空港经济区建设，完善区域基础设施，积极申报临空经济示范区。

　　扩大对外交流合作。落实“双核驱动”战略，继续推进北部湾经济区综合配套改革；深化与珠江—西江经济带沿线城市合作，主动承接珠三角地区产业转移，加快西江基础设施大会战项目建设。积极融入粤桂黔高铁经济带建设。用足用好CEPA先行先试政策，推动一批与港澳合作的项目落地。以国际友城为平台，深化对外交流。

　　加大招商引资力度。深入实施招商引资三年行动计划，大力推进招商引资“六大专项行动”，围绕三大重点工业产业、三大重点服务业和石墨烯、节能环保等新兴产业，以重大项目为抓手，加强产业集群和产业链招商。建立招商项目库、政策库，充分利用标准厂房精准招商，落实招商引资重点项目协调推进和快速落地机制，加速项目落地。

　　提升外向型经济水平。深入实施外经贸发展三年行动计划，大力发展跨境电子商务，完善外贸综合服务平台，加快广西—东盟区域（南宁）外贸一体化综合体通关服务提速工程建设，为外经贸企业提供 “一站式”服务。实施新一轮加工贸易倍增计划，推进高新区等加工贸易发展示范和重点园区建设，加快形成集聚发展优势。扶持外贸龙头企业发展，加强出口品牌建设。提升口岸服务功能，壮大口岸经济。鼓励企业“走出去”，开展对外投资和国际产能合作，促进外经贸形势持续向好。

　　（四）加快打造生态宜居城市

　　坚持治水、建城、为民主线，围绕2018年五象新区核心区基本成型、园博园开园、邕江展新颜，以崭新的城市面貌向自治区60周年大庆献礼，加快推动绿城品质升级，让南宁蓝天常驻、青山常在、碧水常流。

　　打好治水攻坚战。全面推进国家海绵城市试点建设。加快推进54.6平方公里示范区项目及海绵片区建设，海绵城市试点建设三年计划203个项目实现全面开工，竣工率达65%以上，实现示范区整体年径流总量控制率不低于75%、年径流污染控制率不低于50%的目标，努力通过试点验收。深入推进邕江综合治理。推进中心城区55公里岸线整治出效果，实施精品示范段、邕江南岸公园和邕江北岸公园提升工程，开工建设老口航运枢纽—清川大桥、三岸大桥—邕宁水利枢纽段项目。加快推进邕宁水利枢纽建设，争取年底前具备蓄水通航条件，2018年10月完成建设。高标准打造“百里秀美邕江”。基本完成建成区99.4公里黑臭水体整治任务。开工建设黑臭水体整治PPP项目，加快建设沙江河流域综合整治、水塘江、心圩江流域治理等PPP项目工程，整体改造城市内河水系。实施邕江饮用水水源地取水口上移工程。完善邕江上游水源地保护措施，取水口上移进入实质建设阶段，力争2018年工程完工。实行最严格水资源管理制度。抓好中央环保督察反馈意见的整改落实。推进大王滩第二水源地和县区重要水源地保护，保障城乡供水安全。推进水生态文明建设，全面推行河长制，完善水治理体系。

　　提速建设五象新区。加速产城融合，推进中国—东盟电子商务产业园、中国—东盟国际物流基地等园区建设，服务好中国中车、福建吴钢、万达茂等重大产业项目，实现总部基地金融街部分建成使用。加快推进南宁市十四中、三中初中部五象校区和新区第二、第三实验小学等优质学校建设。开工建设广西医科大学东盟国际口腔医学院等卫生项目。加快重点片区路网建设。实现青山大桥等一批桥梁建成通车。加快供水加压站、污水管网、公交场站、垃圾转运站等市政配套设施建设。

　　加快打造“中国绿城”升级版。举全市之力筹办第十二届中国国际园林博览会，按国家5A级景区标准，高水平规划、高标准建设园博园，打造“生态的园博、文化的园博、共享的园博”，确保园博园2018年8月试运营、12月建成开园。深化国家森林城市和国家生态园林城市建设。加快东盟文化博览园建设步伐，努力打造成为文化类型丰富多样、十国文化特色鲜明的现代文化博览园。推进公园“一园一品”建设，打造一批公园和街头小游园，对一批道路进行绿化改造，实施城市精品线路美化亮化彩化提升工程，努力实现居民出行“300米见绿、500米见园”。建设更多的生态廊道、绿色建筑、立体绿化，促进生态与城市相生共融。依法保护利用青秀山、五象岭、大明山等宝贵资源。推进西津、大王滩国家湿地公园试点建设。

　　大力疏解交通拥堵。完善路网体系。开工建设仁义大桥，推进东西向快速路和原有高速公路东环改造工作。整治拥堵节点。开展快速环路综合整治，启动邕武、秀灵—友爱立交改造，新建大学—清川、秀厢—鲁班等立交，加快中华—园湖、凤凰岭—高速环路立交建设，建设和改造一批人行过街天桥设施。打通“断头路”。开展道路建设征地拆迁攻坚战，打通或拓宽一批市区“断头路”“瓶颈路”，重点建设亭洪路延长线、金桥路、降桥路二期、建兴路等，加强城市支路、微循环道路建设，畅通城市交通的“毛细血管”。提高公共交通出行分担率。大力推进轨道交通建设，确保地铁2号线开通试运营，加快推进3、4号线建设，全力推动2号线东延长线和5号线开工建设。加快快速公交（BRT）线网建设，开工建设2号线。建设一批公交场站，完善公交专用道网络，做好与轨道交通1、2号线的接驳工作。完善公共自行车租赁系统和自行车道、步行道等城市慢行交通设施，解决出行“最后一公里”问题。制定、落地和执行网约车新政，规范预约出租汽车，加快出租汽车行业改革。

　　让南宁蓝天常驻。实行最严格的环境保护制度。积极推广天然气、电能、太阳能等清洁能源应用，基本淘汰燃煤小锅炉，加速淘汰黄标车，加强对工业大气污染源、餐炊油烟、机动车尾气排放等的监管与整治。狠抓大气污染治理，提升扬尘污染专项治理成效，严管建筑工地、工程用车、消纳场等，从源头到终端建立标准化、规范化、精细化的大气污染防控长效管理机制，确保空气质量在省会城市保持前列，力争进入全国重点城市前十位。

　　提高城市治理水平。持续推进城市管理精细化、法治化、智慧化、社会化。全面铺开社区网格化治理模式。扎实推进全国综合行政执法体制改革试点。建设“智慧南宁”，按照“一朵云、五平台、多维应用”的技术框架，打造智慧治理、智慧民生、智慧产业、智慧生活、智慧双创五个综合平台，提升医疗、教育、社保、交通、城管等领域的智慧化运用。加快地下综合管廊试点城市建设，建成41.36公里地下综合管廊。开展污水管网普查，老城区开展雨污分流改造试点并逐步推广，新城区新建建筑的排水系统按雨污分流接入对应管网，实现雨污分流率100%。推进江南污水处理厂三期、埌东污水处理厂四期及22个镇级污水处理设施建设，逐步实现污水处理全收集全处理目标。完善桥梁管理“三制”。严厉打击“两违”。加强环卫市场化改革，深入开展“以克论净·深度清洁”活动。开展生活垃圾分类试点工作，推广华润水泥窑协同处理生活垃圾技改项目模式，加快餐厨垃圾无害化处理项目扩容，推进江南循环经济产业园前期工作。重视城市运行安全风险管控工作，加快市防汛抗旱救灾物资储备中心建设。

　　（五）推进供给侧结构性改革和重点领域改革

　　突出问题导向，聚焦重点领域，推出一批重大改革举措，确保改革更加有力有效。

　　深入推进“三去一降一补”。强化行业准入管理，保持重点去产能行业无违规新增产能，扶持企业革新技术装备，全面完成自治区下达的60万吨水泥去产能任务，深入整合木薯淀粉等过剩产能。先期开展具备职工安置条件的30家“僵尸企业”的安置工作，建立非国有“僵尸企业”处置机制。调整商品房供应结构，通过扶持创业、发展健康产业等多种途径化解我市商务办公用房、商业用房库存过高问题。健全完善政府性债务管理体系，积极应对和妥善处置债务风险。调整完善降成本30条措施，全面落实各项减税、免税和停免征部分政府性基金政策，继续推进政府性融资担保体系建设，加大降费、降低要素成本力度，完善降成本的长效机制，切实减轻企业负担。从人民群众迫切需要解决的突出问题着手，补齐公共服务能力、基础设施薄弱环节和生态环境保护的短板。

　　纵深推进重点领域改革。持续推进“放管服”改革。制定出台市县（区）行政审批事项、前置条件等目录，清理不规范的中介服务，全面清理面向公民、法人和其他组织的证照年检、政府指定培训事项。继续推进企业“多证合一、一照一码”和个体工商户“两证整合”以及“双随机、一公开”联合检查等商事制度改革。深化不动产统一登记改革，推进覆盖城乡的不动产统一登记。实现市县（区）权责清单“两单”融合。深化财税体制改革。改进年度预算控制方式，稳步推进中期财政规划编制。研究调整财政事权和支出责任划分，完善财政转移支付制度。深入推进政府购买服务改革，完善政府采购制度。积极落实消费税、资源税等税制改革事项。落实国税、地税综合体制改革任务。扎实推进国企国资改革。做好优化配置、提质增效、防止流失等重点工作，做强做优做大市属国有企业。积极稳妥发展多种形式的混合所有制，抓好国企发展混合所有制试点和职工持股试点工作。推进国有资本投资、运营公司试点。完善法人治理机制，规范董事会建设。推动以管资本为主的国资监管体制建设。深化金融改革创新。深入推进沿边金融综合改革试验区建设，创新跨境人民币业务，加快建设中国—东盟金融信息发布共享平台，探索中国—东盟（南宁）货币指数应用，提升南宁跨境金融信息服务基地功能。支持各类资本发起设立各类法人金融机构，推进金融、科技、产业融合创新发展。创新担保机制，建立“4321”新型政银担风险分担机制。开展农村金融改革，大力发展普惠金融。加强金融监管。

　　（六）推动城乡统筹协调发展

　　优化城市空间布局，做大做强县域经济，全面开展“美丽南宁·宜居乡村”活动，促进城乡协调发展。

　　加快推进新型城镇化。推进城市总体规划修编，完成《南宁市综合交通规划（修编）》，编制《南宁市总体城市设计》等专项规划，落实单元式控制性详细规划与城市设计三年攻坚，加快伊岭工业集中区、隆安华侨管理区、黎塘工业园3个自治区产城互动试点园区建设，推进“多规合一”与规划信息化建设。深化户籍制度改革，完善住房、教育、养老等综合配套改革措施，有序推进农业转移人口市民化。深入实施大县城战略，增强对周边农村地区的带动能力，建设古辣、金陵、古零、伶俐等一批特色小镇。持续推进自治区新型城镇化示范县宾阳县建设，启动第三批国家新型城镇化综合试点镇六景镇试点。

　　推进旧城改造。遵循“成片改造、统一规划，分类实施、总体提升”的原则，按照城市修补和生态修复的要求，创新旧改模式，将旧改与产业结构调整、业态转型升级、城市环境再造相结合，加快推进城市有机更新和棚户区改造步伐。编制实施旧城改造三年滚动计划，提高棚户区改造货币化安置率，积极探索引入法律（律师）服务百姓的征收新模式，重点推进轨道交通沿线、邕江沿岸两侧的成片旧城棚户区改造及“老南宁·三街两巷”改造，打造一批精品项目。

　　发展壮大县域经济。把县域经济发展作为稳增长的重要抓手。夯实县域经济发展基础。完善县域交通网络，重点推进新江至崇左扶绥一级路（南宁段）、张村至六景二级路、大王滩二级路、上林至武鸣二级路、伶俐至陆屋二级路、大塘至渠黎二级路、思陇至那桐二级路、西乡塘005县道改扩建等项目，启动G322国道南宁至宾阳（黎塘）一级路前期工作，推进城乡交通运输一体化建设工程。推进城乡公共服务均等化，加快光网南宁、无线南宁、移动互联网、下一代广播电视网建设，提升互联网城域出口和用户接入能力，加快电网、油气管网等建设，加快推进“县县通”天然气工程。全力打好县域经济翻身仗。结合主体功能区规划，加快推进新型工业化、信息化、城镇化、农业现代化深度融合，引导县域错位发展、多元发展，加快一批特色工业园区、现代特色农业示范区、精品旅游线路建设，着力打造工业大县、农业强县、旅游名县、生态美县、开放活县。推进扩权强县工作，深化县域体制机制改革，统筹推进县改市、乡改镇、村改居。

　　扎实推进宜居乡村建设。重点实施“产业富民”“服务惠民”“基础便民”三个专项活动。推进村级综合服务中心建设，重点做好“村级就业、社会保障经办、教育助学、卫生健康、文化体育、法律”六项服务。综合治理乡村环境，完成自治区下达的农村改厕、改厨和改圈任务，完成农村危房改造8000户；保障农村交通、通信、供电、安全用水、能源利用，推进以病险水库（闸）除险加固、生态清洁河流治理、区域规模化灌区节水配套改造为重点的水利基础设施建设；实施化肥农药使用量零增长行动，加强农业面源污染治理和土壤重金属污染防治。结合地域和民族特色，抓好历史文化名镇、传统村落和古树名木保护，秉承乡土理念开展第二批49个乡土特色示范村建设，完成列入中国传统村落名录江南区扬美村等4个村建设，传承文化，留住乡愁。

　　（七）继续推进精准脱贫攻坚

　　按照“小康路上一个都不能掉队”的要求，深入实施 “七个一批”“七大工程”，全面完成自治区下达的年度脱贫攻坚任务。

　　集中攻关重点难点。把产业扶贫摆在第一位，把贫困群众受益摆在中心位置，建立完善市县乡村四级脱贫攻坚项目库，列出项目清单；按照“合作社（龙头企业）+经济能人”等模式，大力发展特色种养业、乡村旅游业、农村电商；大力发展村集体经济，建立健全贫困户参与机制和受益机制，扶持每个预脱贫村成立至少1个农民专业合作社。稳步推进贫困村屯级道路、水利、农网改造等公共服务设施建设。实施16个易地扶贫搬迁安置点建设。

　　增强贫困户后续发展能力。推进教育扶贫，加大贫困地区农村学校教育投入，落实对建档立卡贫困户子女从学前教育到高中阶段15 年免费教育的资助政策。加强就业创业和职业技能培训，开发乡村公益性岗位，加快贫困劳动力转移就业，扶持贫困劳动力创业致富。加大健康帮扶力度，对农村贫困人口住院治疗实行先诊疗后付费，对因病致贫、因病返贫建档立卡贫困户人口实施分类救治，健全城乡居民基本医疗保险、城乡居民大病保险衔接机制。狠抓后续扶持，推动稳定脱贫。

　　增强精准脱贫攻坚实效。突出县区主体责任，抓好责任落实、政策落地和群众动员三个关键环节。严格“一把手”脱贫攻坚责任制和“四到县”制度，强化干部定点挂钩、对口帮扶，健全结对帮扶到村到户“三包”机制。坚持督查、通报、协调三项制度，切实抓好绩效考核和第三方评估工作。落实产业、金融等扶贫政策，整合各类扶贫资金，提高扶贫资金使用的精准性、有效性。制定脱贫激励措施，发挥群众主观能动性，注重调动基层积极性，坚决克服“干部干、群众看”的现象。严格执行“一户一册一卡”，落实脱贫摘帽退出机制，完善市级脱贫攻坚信息大数据管理平台，实行动态、精确、全面管理，严防“数字脱贫”。推动社会扶贫资源与扶贫需求的精准对接，形成扶贫合力。

　　（八）坚持保障和改善民生

　　以增进人民福祉为出发点和落脚点，破解民生难题、回应民生关切、满足民生需求，提升人民群众获得感。

　　加强就业和社会保障工作。继续实施就业优先战略，统筹抓好高校毕业生和困难群众就业，支持返乡农民工创业就业。严厉打击拖欠农民工工资行为，保障农民工合法权益。整合城乡居民基本医疗保险制度，扩大社会保险覆盖范围。完善重特大疾病医疗救助制度，稳步提高困难群众的医疗救助水平。提升城乡低保保障能力，实现应保尽保。落实特困人员救助供养制度，提高特困人员供养水平。加快市第二社会福利院建设。健全住房保障体系，狠抓保障性住房基本建成率，提高分配入住率。

　　提升教育发展水平。加快教育管理体制改革，开展义务教育学区制改革和集团化办学，推动教育管办评分离。编制第三期学前教育行动计划，大力发展公办幼儿园，新建成公办幼儿园12所，鼓励多元普惠幼儿园发展。新建成中小学校8所，改扩建一批中小学校。力争5个县区通过义务教育均衡发展自治区督导评估。推动普及高中阶段教育，促进普通高中多样化发展，提升高中教育教学质量水平。强化职业院校校企合作，重点培养适合我市产业发展的高技能人才。统筹安排进城务工人员随迁子女入学。支持和规范民办教育发展。加快南宁教育园区基础设施建设，安排项目24个、投资23.2亿元。加强市属高校建设，启动南宁学院升格为南宁大学的基础性工作。扩大教育对外交流合作，推进国际学校建设。

　　抓好卫生与健康工作。突出抓好城市公立医院综合改革、实施分级诊疗、完善城乡医疗卫生服务体系建设等重点工作。整合卫生计生资源，探索建立区域医疗联合体。持续深化县级公立医院综合改革及基层医改。推进智慧健康信息工程项目（一期），建立市级电子健康档案和电子病历数据库平台，实现13家市直属医院病历资料、检查结果等信息互通互认。开展健康城市建设和区域影像诊断中心应用平台建设试点。扶持中西医、壮瑶医事业发展。加强全科医生、产科、儿科医生等骨干人才培养。平稳实施全面两孩政策，改善出生人口性别结构，加强优生优育指导，促进人口均衡发展。

　　繁荣文化事业壮大文化产业。策划、创作和推出一批内容深刻、制作精良的文艺精品，讲好南宁故事。精心打造“天下民歌眷恋的地方”亮丽名片，力争将“三月三”打造成民族文化嘉年华。继续打造民歌湖“百姓大舞台”周周演群众文化活动品牌。积极推进国家历史文化名城申报，建成广西文化艺术中心，启动顶蛳山遗址保护工程。改版升级“春天的旋律”跨国春晚，精心组织中国—东盟（南宁）戏剧周、“文化走亲”东盟行等活动，扩大南宁文化影响力和吸引力。推进落实“十三五”文化产业发展规划，发展“一带两区三组团”文化产业新格局，培育特色文化产业集聚区。深化文艺院团改革，增强文化表演院团造血功能和适应市场能力。

　　加快发展体育事业。深入实施《南宁市全民健身实施计划（2016－2020）》，抓好竞技体育，加快推进市体育运动学校一期建设。建设全民健身“互联网+”平台，提升全民健身服务水平。加快发展体育产业，促进体育与旅游深度融合。积极申办“苏迪曼”杯世界羽毛球混合团体锦标赛，办好“中国杯”国际足球锦标赛、环广西公路自行车世界巡回赛等重大赛事。

　　持续加强社会治理创新。积极推进社区治理和服务创新，开展社区协商，推行社区公共服务事项准入制度。深入推进平安南宁建设，扎实推进“智慧警务”项目建设，加快构建立体化、信息化的社会治安防控体系，严厉打击电信诈骗等违法犯罪活动。进一步落实“法治信访、阳光信访、责任信访”，畅通信访渠道，加强涉访涉诉渠道建设。建立健全安全风险分级管控和隐患排查治理双重预防性工作机制，坚决防范重特大公共安全事故发生。在全市开展“诚信经营、放心消费”创建工作，重点打造放心消费示范店、示范街、示范商圈。健全食品药品安全监管体制，积极创建国家食品安全城市。健全应急管理体制机制，提高综合防灾减灾救灾能力。建立健全市县（区）乡（镇、街道）三级公共法律服务体系运转机制。支持军队改革，抓好优抚安置政策落实，促进军民深度融合发展。巩固全国民族团结进步示范市创建成果。做好村（社区）“两委”换届工作。

　　坚持为民办实事。坚持财政支出更多向民生倾斜，全力推进教育、食安、健康等10大项为民办实事工程，让发展成果更多惠及百姓。

　　三、全面加强政府自身建设

　　坚决维护习近平总书记的核心地位，把守纪律讲规矩摆在更加重要的位置，切实增强“四个意识”特别是核心意识、看齐意识，努力营造风清气正的政治生态，用新理念引领新发展，以新状态干出新成效。

　　建设法治政府。认真履行党政主要负责人推进法治建设第一责任人职责，大力推进政府立法公开透明，拓宽公众参与立法渠道，建立法规规章起草征求人大代表意见制度和立法协商制度，更多引入第三方参与政府立法的起草、论证、评估等工作，建立一批立法基层联系点。实行规范性文件“三统一”制度，强化规范性文件备案审查，全面清理规章和规范性文件。运用网上行政执法暨电子监察系统强化对行政处罚的实时监督，规范执法辅助人员管理，促进严格规范公正文明执法。加强基层法制机构和队伍建设，建立乡镇法制员队伍，使法制机构、人员与其承担的职能相匹配。深入开展“七五”普法，弘扬宪法精神，引导全社会尊法、学法、守法、用法。

　　坚持科学决策。严格执行重大行政决策征求意见、咨询论证、风险评估、合法性审查、集体讨论决定等程序，增加公众参与重大行政决策实效，确保决策科学、民主、合法。完善政府法律顾问制度，健全行政决策机制。主动接受市人大及其常委会法律监督和工作监督，认真办理人大代表议案建议。自觉接受市政协民主监督，健全重大事项事前协商制度，认真办理政协提案。

　　强化作风建设。巩固“两学一做”学习教育成果，促进政府机关作风持续转变。持之以恒落实中央八项规定精神，严守国务院“约法三章”，及时处置懒政怠政现象，严防“四风”反弹，严格控制“三公”经费支出，进一步规范办公用房的建设使用。严格履行党风廉政建设“一岗双责”。加强审计监督，深化领导干部经济责任审计，加大对公共资金、公共资源、国有资产的审计力度。加强公共资源交易监管，确保公共资源交易活动公开公平公正、高效规范廉洁。完善督促检查工作机制，建立健全激励机制和容错纠错机制，激发干部干事创业积极性。

　　优化政府服务。用好用活用足中央、自治区和我市出台的系列政策。大力推进“互联网+政务服务”，完善市网上审批大厅预受理服务和办件预约功能，力争实现80%的政务服务事项可上线申报，让企业和群众少跑腿、好办事。全面清理涉及群众办事的各种不必要证明。充分发挥市行政审批局作用。抓好相对集中行政许可权试点改革。以权责清单为基础，积极探索审批流程再造，切实提高政务服务效能，减少审批环节，压缩承诺办结时间。完善市政府例行新闻发布制度。强化各级政府网站建设和管理，推动决策、执行、管理、服务和结果“五公开”，做好政务舆情回应，打造阳光政府。

　　各位代表！肩负新使命，实现新目标，关键在于落实。让我们紧密团结在以习近平同志为核心的党中央周围，在自治区党委、政府和市委的坚强领导下，以更加坚定的信心、更加昂扬的斗志、更加扎实的作风，撸起袖子加油干，为首府加快建设“四个城市”，勇当广西营造“三大生态”、实现“两个建成”的排头兵作出新的更大贡献，以优异成绩迎接党的十九大胜利召开！
